# Regulation of VISA/MAVS Signalosome Dynamics and Immune Homeostasis

**DOI:** 10.3390/biology15151224

**Published:** 2026-07-23

**Authors:** Qi-Peng Shu, Shang-Ze Li

**Affiliations:** 1 School of Medicine, Chongqing University, Chongqing 400030, China; qipengshu@cqu.edu.cn; 2 College of Medicine, Tibet University, Lhasa 850000, China

**Keywords:** VISA/MAVS, innate immunity, immune homeostasis, activation, termination and restriction, viral infection

## Abstract

VISA/MAVS is a central signaling hub that links viral RNA sensing to type I interferon production and inflammatory responses. Because of its potent signal-amplifying capacity, VISA activity must be precisely controlled: insufficient activation compromises antiviral defense, whereas excessive or spontaneous activation can drive chronic inflammation and autoimmune disease. Recent studies have revealed a complex regulatory network governing VISA signaling, involving post-translational modifications, dynamic protein interactions, selective degradation pathways, metabolic cues, and intrinsic inhibitory mechanisms that collectively determine its activation threshold, signaling duration, and downstream output. In this review, we discuss the molecular mechanisms that regulate VISA activation, signal propagation, signal termination, and quiescence maintenance under resting conditions. We propose that immune homeostasis is achieved not through a simple on–off switch, but through continuous regulation of the VISA signalosome life cycle. This framework provides an integrated view of VISA and offers new insights into the molecular mechanisms underlying immune homeostasis.

## Simple Summary

The innate immune system must rapidly respond to viral infections while preventing unnecessary immune activation that can damage healthy tissues. A key protein called VISA acts as a molecular switch that controls this process. In this review, we describe how VISA is dynamically regulated throughout its signaling life cycle—from activation during viral infection to signal termination and maintenance of a resting state under normal conditions. By presenting these regulatory mechanisms within a single framework, we highlight how precise regulation of this signaling pathway maintains immune homeostasis throughout the VISA signaling life cycle.

## 1. Introduction

Virus-induced signaling adaptor (VISA) [1], also known as mitochondrial antiviral-signaling protein (MAVS) [2], is a central signaling hub that orchestrates innate immune responses against viral infection [3,4]. VISA is predominantly localized on the outer membrane of mitochondria, as well as on mitochondria-associated membranes and peroxisomes [5,6]. VISA serves as the indispensable adaptor linking cytosolic viral RNA sensing by RIG-I-like receptors (RLRs) to the activation of downstream antiviral and inflammatory transcriptional programs [1].

**Figure 1:**
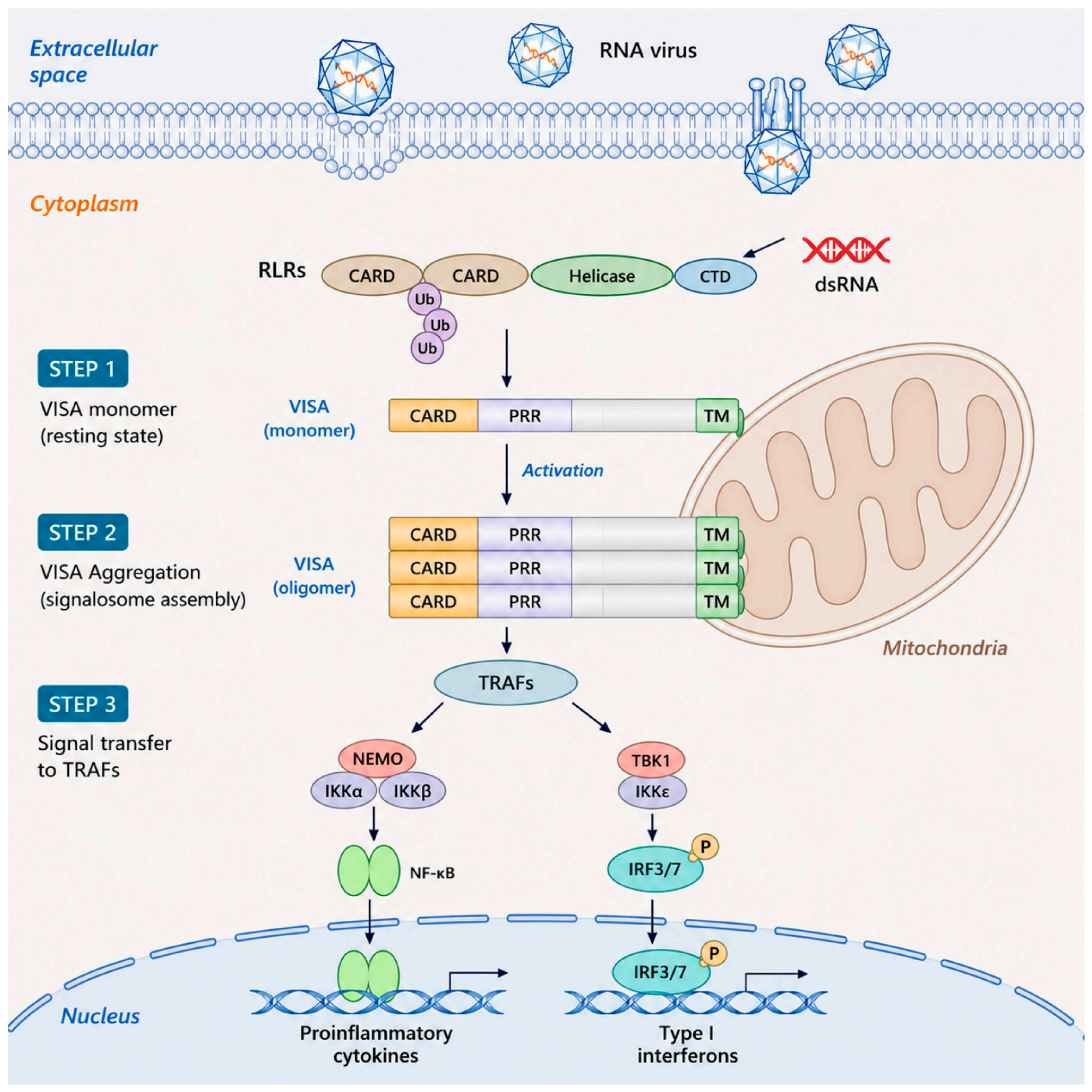
VISA signaling transduction. **STEP 1:** Under resting conditions, VISA is maintained in an inactive monomeric state by multiple regulatory mechanisms that prevent spontaneous activation. **STEP 2:** Upon recognition of viral RNA, activated RIG-I-like receptors (RLRs) engage mitochondrial VISA through CARD–CARD interactions, triggering prion-like aggregation of VISA into higher-order signalosomes. **STEP 3:** Aggregated VISA signalosomes recruit TRAF family adaptor proteins, which activate the TBK1–IKKε and IKKα/IKKβ–NEMO signaling cascades, leading to phosphorylation of IRF3/7, activation of NF-κB, and subsequent induction of type I interferons and pro-inflammatory cytokines.

Upon recognition of viral RNA, activated RLRs engage VISA through CARD–CARD interactions [1], triggering the rapid conversion of monomeric VISA into self-propagating prion-like aggregates [7]. These higher-order signalosomes subsequently recruit multiple downstream signaling components, including tumor necrosis factor receptor-associated factors (TRAFs) [8,9], TANK binding kinase 1 (TBK1) [10], and IκB kinase complex (IKK complexes) [8], culminating in the activation of Interferon Regulatory Factor 3 (IRF3) [11], Interferon Regulatory Factor 7 (IRF7) [3], and Nuclear Factor kappa-B (NF-κB) [8,12,13] and the induction of type I interferons (IFNs) and pro-inflammatory cytokines [14,15] (Figure 1).

The extraordinary signal-amplifying capacity of VISA aggregation endows the host with a powerful mechanism for rapid antiviral defense [7,16]. However, this amplification mechanism also creates an inherent challenge: VISA signaling must be robustly activated upon pathogen encounter yet efficiently terminated once the threat is eliminated. Excessive or prolonged VISA activation can drive uncontrolled inflammatory responses, contributing to cytokine storms, tissue injury, and chronic immunopathology. Equally detrimental, spontaneous VISA aggregation in the absence of infection can break immune tolerance and promote aberrant type I IFN production, a hallmark of numerous autoimmune and autoinflammatory disorders, including systemic lupus erythematosus and psoriasis [17,18,19,20,21,22]. Consequently, the assembly, maintenance, and disassembly of VISA signalosomes are subject to multilayered regulatory mechanisms operating at both basal and activated states.

Previous reviews have primarily summarized VISA regulation according to individual classes of post-translational modifications or specific signaling molecules. Although these studies have substantially advanced our understanding of VISA biology, they often discuss individual regulatory events in isolation, making it difficult to appreciate how distinct regulatory mechanisms are coordinated throughout the signaling process. Here, rather than categorizing regulators by modification type, we organize the current knowledge according to the dynamic life cycle of the VISA signalosome, encompassing signal initiation, amplification, termination, and quiescence maintenance. This perspective highlights how multiple layers of post-translational regulation cooperate to define the activation threshold, signaling duration, and eventual restoration of immune homeostasis.

Furthermore, this framework allows diverse regulatory mechanisms, including post-translational modifications, translational regulation, alternative isoforms, and selective protein degradation, to be interpreted within a unified conceptual model rather than as isolated regulatory events.

## 2. Mechanisms of VISA Activation During Viral Infection

### 2.1. Viral RNA Sensor-Mediated VISA Activation

Activation of innate immune responses is initiated by the recognition of pathogen-associated molecular patterns (PAMPs) by pattern-recognition receptors (PRRs) [23]. Among the major PRR families, including Toll-like receptors (TLRs), RIG-I-like receptors (RLRs), NOD-like receptors (NLRs), and cytosolic DNA sensors [24], RLRs serve as the principal detectors of RNA virus infection. Upon viral invasion, viral RNA is recognized by the cytosolic RNA sensors RIG-I and MDA5 [3,25]. Both RIG-I and MDA5 contain tandem N-terminal caspase recruitment domains (CARDs), which engage the CARD domain of VISA through homotypic CARD–CARD interactions, thereby initiating VISA aggregation [3,4,7].

Beyond the canonical RLR pathway, several non-canonical RNA sensors have also been shown to converge on VISA-dependent signaling. NOD2 directly recognizes viral single-stranded RNA and subsequently utilizes VISA to activate IRF3 [26]; NLRP6 cooperates with DHX15 to sense viral RNA and induce type I interferons (IFNs) and interferon-stimulated genes (ISGs) through VISA [27]. In addition, ZNFX1 and GSDMB function as viral RNA sensors that interact with VISA to trigger type I IFN production [28,29]. Notably, recent evidence suggests that VISA itself can directly bind viral RNA, thereby contributing to antiviral signaling independently of upstream RNA sensors [30].

As the central adaptor of the RLR pathway, VISA transduces antiviral signals through the formation of higher-order prion-like aggregates [7]. Following viral RNA recognition, activated RIG-I and MDA5 undergo CARD oligomerization, generating multivalent CARD assemblies that nucleate VISA CARD filament formation via homotypic CARD–CARD interactions [31,32,33]. This nucleation event drives the rapid conversion of monomeric VISA into self-propagating prion-like polymers, leading to the assembly of functional VISA signalosomes and the initiation of downstream antiviral signaling [34].

Collectively, these findings establish VISA as a central signaling hub that integrates inputs from both canonical and non-canonical viral RNA sensors. Although RIG-I and MDA5 remain the primary initiators of VISA activation during RNA virus infection, emerging evidence suggests that multiple RNA-sensing pathways can converge on VISA to trigger antiviral signaling. Through this convergent architecture, VISA serves as a molecular checkpoint that transforms diverse viral recognition events into a unified signalosome assembly program, thereby ensuring robust and coordinated antiviral responses.

### 2.2. Activation of VISA Aggregation

In addition to receptor-mediated activation, post-translational modifications (PTMs) play indispensable roles in regulating VISA aggregation (Table 1). A growing body of evidence indicates that multiple PTMs, including K63-linked ubiquitination [35,36], deacetylation [37], SUMOylation [38], and phosphorylation [39], collectively facilitate the transition of VISA from an inactive monomeric state to an aggregation-competent signaling platform. Among these modifications, K63-linked ubiquitination represents one of the best-characterized mechanisms promoting VISA aggregation and signalosome assembly.

Recent studies have further highlighted an emerging connection between cellular metabolism and antiviral innate immunity. Metabolite-derived modifications have been shown to fine-tune VISA activation by modulating its aggregation propensity. For example, O-GlcNAc transferase (OGT)-mediated O-GlcNAcylation of VISA enhances K63-linked ubiquitination, thereby facilitating VISA aggregation and downstream signaling [40]. Likewise, palmitoylation has recently emerged as a critical regulatory mechanism controlling VISA activation. Several palmitoyl transferases, including ZDHHC24 [41], ZDHHC4 [42], ZDHHC12 [43], and ZDHHC7 [44], directly catalyze the palmitoylation of VISA at distinct cysteine residues, promoting its prion-like aggregation and antiviral activity during viral infection.

Beyond PTM-mediated regulation, several post-translational mechanisms have also been shown to facilitate VISA activation independently of direct covalent modification. These regulatory events primarily operate through remodeling protein–protein interactions or altering the molecular environment that favors VISA signalosome assembly. For example, Met has been reported to directly associate with VISA and promote signalosome formation, thereby enhancing the production of antiviral cytokines [45]. In addition, FZR1 facilitates VISA aggregation by mediating degradation of the glycolytic rate-limiting enzyme PFKFB3, highlighting a previously unrecognized link between cellular metabolic programs and VISA signalosome dynamics [46]. These findings suggest that, beyond serving as substrates for PTMs, VISA signalosomes are also regulated by dynamic protein interaction networks and metabolic cues that collectively shape their activation potential.

Collectively, these studies demonstrate that the transition of VISA from an inactive monomer to an aggregation-competent signalosome is governed by a multilayered post-translational regulatory network. Beyond the initial nucleation event induced by upstream RNA sensors, diverse PTMs and PTM-independent regulatory mechanisms cooperate to enhance, stabilize, and amplify VISA aggregation. An emerging theme is that although diverse upstream regulators employ distinct biochemical mechanisms, most ultimately converge on promoting VISA aggregation, highlighting aggregation as the central regulatory node governing antiviral signaling. Another notable trend is the increasing integration of metabolic enzymes and metabolite-derived modifications into VISA regulation, suggesting that antiviral signaling is closely coupled with cellular metabolic adaptation rather than functioning as an isolated immune pathway. Thus, these regulatory layers ensure that VISA aggregation occurs efficiently and reaches a threshold sufficient to initiate potent antiviral responses.

### 2.3. Stabilization of VISA Protein

The intensity and duration of VISA-mediated antiviral signaling are determined not only by the efficiency of signalosome assembly but also by the abundance and stability of the VISA protein. Although prion-like aggregation is required for signal initiation, sustained antiviral responses rely on maintaining sufficient levels of VISA to support signalosome integrity and downstream signaling. Consistent with this notion, VISA undergoes progressive degradation during the late stages of viral infection, suggesting that stabilization of VISA represents an additional regulatory mechanism for amplifying and prolonging antiviral signaling.

To date, VISA turnover has been shown to occur predominantly through ubiquitin-dependent selective degradation pathways. Consequently, mechanisms that antagonize degradative ubiquitination can preserve VISA abundance and enhance antiviral signaling. For example, OTUD4 removes K48-linked polyubiquitin chains from VISA, thereby preventing its proteasomal degradation and promoting type I interferon production [47]. Similarly, PCSK9 and ATP13A1 have been reported to enhance innate immune responses by protecting VISA from proteasome-mediated turnover [48,49]. In addition to regulating VISA activity, notably, ZDHHC4-mediated palmitoylation of VISA at C79 can also stabilize the protein and support sustained antiviral signaling [42].

In summary, these findings suggest that the antiviral output of VISA is governed by two interdependent parameters: signalosome assembly and protein abundance. Whereas aggregation initiates signal transduction, stabilization of VISA sustains signalosome function and prolongs downstream antiviral responses. Thus, mechanisms that preserve VISA stability represent an important layer of regulation controlling both the intensity and duration of innate immune signaling.

### 2.4. Recruitment of Downstream Signaling Molecules

Following signalosome assembly, VISA functions as a molecular scaffold that orchestrates the recruitment and activation of downstream signaling complexes. Through its proline-rich region (PRR), VISA engages multiple TRAF family members, including TRAF2, TRAF3, TRAF5, and TRAF6, thereby establishing a signaling platform for the propagation of antiviral responses [8,50]. The assembly of these TRAF-containing complexes facilitates the recruitment and activation of the downstream kinases TBK1 and IKKε, as well as the NEMO-containing IKK complex, ultimately leading to the activation of IRF3/7 and NF-κB. Activated IRF3/7 and NF-κB subsequently translocate into the nucleus, where they drive the transcription of type I interferons and pro-inflammatory cytokines, thereby establishing an effective antiviral state [51,52].

Recent studies have further revealed that the efficiency of downstream signal propagation is subject to additional regulatory control. For example, TRIM21 promotes the interaction between VISA and TBK1 through K27-linked ubiquitination, thereby facilitating downstream antiviral signaling [53]. This process can be further enhanced by UBL7, which strengthens the VISA–TBK1 signaling axis [54]. Moreover, phosphorylation of VISA by TBK1 at the conserved pLxIS motif has emerged as a critical step for efficient IRF3 recruitment and activation [11]. These findings suggest that VISA not only serves as a platform for signalosome assembly but also actively coordinates the spatial and temporal organization of downstream signaling complexes to ensure robust antiviral responses.

Taken together, the activation of VISA during viral infection is a highly coordinated and tightly regulated process. Viral RNA recognition by multiple upstream sensors initiates VISA activation, while diverse post-translational modifications and regulatory mechanisms promote signalosome assembly and maintain VISA stability. These events collectively facilitate the recruitment and activation of downstream signaling complexes, ultimately leading to the robust induction of type I interferons and pro-inflammatory cytokines. Thus, efficient antiviral signaling relies not only on the initiation of VISA aggregation but also on the precise orchestration of signalosome maturation, stabilization, and downstream effector engagement. The dynamic regulation of these processes enables rapid antiviral defense while providing multiple checkpoints for subsequent signal termination and restoration of immune homeostasis.

## 3. Mechanisms Terminating VISA Signaling

The rapid assembly of VISA signalosomes is essential for mounting effective antiviral immune responses. However, the same signal-amplifying mechanism that enables robust production of type I interferons (IFNs) and inflammatory cytokines also poses a substantial risk to immune homeostasis if left unchecked. Excessive, prolonged, or constitutive activation of VISA signaling can result in persistent IFN production, chronic inflammation, tissue injury, and the development of autoimmune and autoinflammatory disorders [17]. Therefore, once antiviral responses have been initiated, VISA signalosomes must be efficiently restrained and ultimately dismantled to restore cellular homeostasis.

Accumulating evidence suggests that termination of VISA signaling is not mediated by a single regulatory event but instead involves a multilayered control network operating at distinct stages of the signalosome life cycle. These mechanisms can be broadly categorized into three regulatory layers: inhibition of VISA aggregation, selective degradation of VISA, and disruption of downstream signaling complex assembly. Through the coordinated action of these regulatory pathways, cells ensure the timely attenuation of antiviral signaling while preventing excessive immune activation and immunopathology.

### 3.1. Inhibition of VISA Aggregation

Given that prion-like aggregation represents the central activation event of VISA signaling, disruption of the VISA signalosome constitutes one of the most effective mechanisms for signal termination. Current evidence indicates that the disassembly of VISA signalosomes can be achieved through both PTM-dependent and PTM-independent regulatory pathways (Table 2).

Notably, several activating modifications described in Table 1 are reversible. Whereas K63-linked ubiquitination, palmitoylation, and SUMOylation promote VISA aggregation and signalosome assembly, their corresponding counteracting modifications, including deubiquitination [36], deSUMOylation [38,55], and depalmitoylation41, effectively reverse VISA activation and suppress downstream antiviral signaling. In addition, SIRT5-mediated desuccinylation of K7 [37], PRMT7-mediated monomethylation of VISA at R52 [56,57], PRMT9-mediated methylation of VISA at R41/43 [58], and lactylation [59] of VISA can also disassociate the aggregation of VISA.

Beyond PTM-mediated regulation, several proteins have been identified as negative regulators of VISA aggregation. WDR77 [60], SAMHD1 [61], TAX1BP1 [62], MAVI1 [63], and NLRX1 [64] inhibit VISA signalosome formation through distinct mechanisms independent of direct covalent modification. Although these regulators employ diverse molecular strategies, they ultimately converge on a common outcome: preventing or dismantling higher-order VISA assemblies and thereby attenuating antiviral signaling.

Collectively, these findings highlight the highly dynamic and reversible nature of VISA signalosome assembly. Rather than representing an irreversible activation event, VISA aggregation is continuously monitored and counterbalanced by multiple inhibitory mechanisms. The coordinated interplay between aggregation-promoting and aggregation-disrupting pathways enables precise control of VISA signaling, ensuring efficient antiviral responses while preventing excessive immune activation.

### 3.2. Selective Degradation of VISA

In addition to disrupting signalosome assembly, selective degradation of VISA represents a major mechanism for terminating antiviral signaling. Given the potent signal-amplifying capacity of VISA aggregates, efficient clearance of VISA is essential to prevent prolonged or excessive type I interferon production, which may otherwise lead to tissue damage and immune-mediated pathology. To date, ubiquitination has been shown to serve as the principal signal directing selective degradation of VISA (Table 3).

Distinct E3 ubiquitin ligases regulate VISA turnover through different ubiquitin linkages and degradation routes. K48-linked ubiquitination primarily targets VISA for proteasomal degradation [22,65,66,67,68,69,70,71,72,73,74,75,76,77,78,79], whereas K27-linked ubiquitination directs VISA toward autophagy–lysosome-mediated clearance [80,81,82]. These findings suggest that cells employ multiple degradative systems to ensure the efficient removal of activated VISA signalosomes.

Although proteasomal and autophagy–lysosome pathways are mechanistically distinct, they function as complementary degradation systems that ensure efficient dismantling of activated VISA signalosomes and restoration of immune homeostasis. Recent studies further suggest that upstream post-translational regulators frequently act as molecule switches that determine the degradative fate of VISA (Table 4). For example, PCBP1/2 [68,69], TAX1BP1 [70], MST4 [75], Ndfip1 [76], OTUD1 [77], PB1 [80], and HFE [83] promote VISA degradation by facilitating the recruitment or activity of distinct E3 ubiquitin ligases, thereby directing VISA toward proteasomal or autophagy–lysosome-mediated degradation. Furthermore, several post-translational modifications, including PARylation [74], palmitoylation [84], and phosphorylation [85,86], have been shown to facilitate the recruitment of degradation machinery or to promote the engagement of specific degradative pathways. Together, these findings indicate that VISA turnover is an actively programmed process rather than a passive consequence of signal attenuation, allowing activated VISA to be selectively eliminated once antiviral signaling has fulfilled its protective function.

In summary, these studies highlight selective degradation as a fundamental mechanism governing the life cycle of VISA signalosomes. Beyond simply removing signaling components, controlled VISA turnover enables the timely resolution of antiviral responses and prevents the detrimental consequences of persistent interferon signaling. Thus, degradation represents a critical checkpoint linking signalosome disassembly to the maintenance of immune homeostasis.

### 3.3. Disruption of Downstream Signaling

In addition to inhibiting signalosome assembly and promoting VISA degradation, antiviral signaling can also be terminated by disrupting the recruitment of downstream signaling components. Unlike mechanisms that dismantle VISA signalosomes, these regulatory pathways attenuate signal transduction by uncoupling VISA from its downstream effectors, thereby preventing the propagation of antiviral signals.

Several negative regulators have been identified that interfere with the assembly of VISA-associated signaling complexes. For example, PPM1A suppresses antiviral signaling by dephosphorylating VISA and impairing its interaction with TBK1 [87]. Similarly, PLK1-mediated phosphorylation of VISA inhibits the recruitment of TBK1 and IRF3, thereby attenuating downstream signal transduction [88]. In addition, PWWP3A has been reported to disrupt the VISA–TBK1 interaction [89], whereas MST4 also inhibits the association between VISA and TRAF3, thereby preventing the efficient activation of downstream signaling pathways [75]. These findings suggest that signal termination can be achieved without dismantling the VISA signalosome itself. By selectively disrupting critical protein–protein interactions within the signaling complex, cells establish an additional regulatory checkpoint that restricts antiviral signaling while preserving the structural integrity of VISA assemblies.

Taken together, these studies reveal that VISA signal termination is not governed by a single inhibitory mechanism but instead relies on coordinated regulation of signalosome dynamics. By controlling VISA aggregation, turnover, and downstream signaling competence, cells precisely define the duration and magnitude of antiviral responses. The integration of these regulatory checkpoints ensures the timely resolution of innate immune signaling and safeguards immune homeostasis following viral infection.

## 4. Maintaining VISA Quiescence Under Resting Conditions

Persistent activation of type I interferon (IFN-I) signaling is widely recognized as a major driver of autoimmune and inflammatory diseases [17,18]. As the central adaptor responsible for antiviral IFN-I production, aberrant activation of VISA has increasingly been implicated in amplifying these pathological immune responses. In patients with systemic lupus erythematosus (SLE), spontaneous prion-like aggregation of VISA has been observed in peripheral blood mononuclear cells and is associated with elevated IFN-I signatures [19]. In addition, mechanistic studies using patient-derived monocytes and lupus mouse models further demonstrated that activation of the RIG-I–VISA pathway amplifies lupus-associated inflammation through coordinated induction of IFN-I and IL-1β [20]. Similarly, studies in psoriasis patients have demonstrated that persistent VISA activation promotes IFN-β production and skin inflammation [21], while psoriasis-associated CARD14 mutants further enhance VISA activation in keratinocytes [22]. Collectively, these findings suggest that persistent VISA activation functions as an important amplifier of autoimmune and inflammatory responses, underscoring the necessity of maintaining VISA in a quiescent state to preserve immune homeostasis.

To prevent spontaneous signalosome formation, cells have evolved multiple mechanisms that directly restrain VISA aggregation. Several naturally occurring truncated VISA isoforms act as dominant-negative regulators that associate with full-length VISA and prevent its spontaneous aggregation [90]. In parallel, an intrinsic autoinhibitory mechanism has been identified in which the TRAF-interacting motifs of VISA are masked by adjacent regulatory regions, thereby limiting downstream signaling complex assembly in resting cells [91]. In addition, PRMT7 and TAX1BP1 have been reported to suppress spontaneous VISA activation by inhibiting VISA aggregation and signalosome formation [58,62].

Beyond controlling aggregation, restricting VISA abundance represents another critical layer of immune homeostasis. Both upstream open reading frames (uORFs) [92] and the E3 ubiquitin ligase RNF115 [72] function to limit basal VISA expression under resting conditions. Importantly, excessive accumulation of VISA can overwhelm intrinsic inhibitory mechanisms and drive spontaneous aggregation even in the absence of viral infection [92]. These observations suggest that maintaining an appropriate threshold of VISA expression is equally important as preventing its aggregation, highlighting the dual requirement for quantitative and qualitative control of VISA activation.

These mechanisms indicate that preventing spontaneous VISA activation is not achieved by a single inhibitory factor but rather by multiple regulatory layers that simultaneously restrict VISA abundance, aggregation propensity, and signaling competence. Together, these regulatory mechanisms ensure that VISA remains poised for rapid antiviral responses while preventing inappropriate activation that would otherwise compromise immune homeostasis.

Interestingly, recent studies have further linked VISA homeostasis to mitochondrial integrity and cellular metabolism. Excessive expression or constitutive activation of VISA can induce mitochondrial damage and trigger mitophagy [90,92], suggesting that uncontrolled antiviral signaling imposes a substantial metabolic burden on host cells. Therefore, maintenance of VISA quiescence serves not only to prevent aberrant interferon production but also to preserve mitochondrial fitness and cellular energetic capacity. By simultaneously constraining VISA aggregation and abundance, cells establish a homeostatic state that minimizes immunopathology while maintaining the ability to rapidly mount antiviral responses upon pathogen encounter.

## 5. Conclusions

VISA has long been recognized as a central signaling adaptor that bridges viral RNA sensing to the induction of type I interferons and inflammatory responses. However, accumulating evidence indicates that VISA is far more than a simple signaling scaffold. Rather, it functions as a highly dynamic signalosome whose activity is continuously regulated throughout its entire life cycle. From receptor-triggered aggregation and signalosome assembly to downstream signal propagation, selective degradation, and maintenance of quiescence under resting conditions, multiple regulatory mechanisms cooperate to precisely control the magnitude, duration, and threshold of VISA signaling.

Collectively, the studies discussed in this review support a conceptual framework in which VISA regulation is orchestrated through a continuous and hierarchical signalosome life cycle. Rather than representing isolated post-translational events, regulatory mechanisms acting at different stages are functionally interconnected. Early activation events establish the signaling threshold, and downstream amplification determines signaling output, whereas selective degradation and quiescence-maintaining mechanisms ensure timely signal resolution and restoration of immune homeostasis. This life-cycle perspective provides an integrated framework for understanding how antiviral signaling achieves both robustness and self-limitation.

Despite substantial progress, several important questions remain unresolved. Future studies are needed to elucidate how distinct regulatory mechanisms are hierarchically and temporally coordinated across different stages of the VISA signalosome life cycle, how metabolic cues are integrated into this regulatory network, and how dysregulation of specific stages contributes to human inflammatory and autoimmune diseases. Addressing these questions will deepen our understanding of innate immune regulation and provide a stronger mechanistic foundation for future studies investigating VISA-associated diseases.

Beyond summarizing current knowledge, this framework of VISA provides a conceptual basis for future investigations into the dynamic regulation of innate immune signaling.

## 6. Literature Search Strategy

This review was conducted as a narrative literature review. Relevant publications were primarily retrieved from the PubMed database using the keywords “VISA”, “MAVS”, “post-translational modification”, “aggregation”, “degradation”, “innate immunity”, “viral infection”, “SLE”, “psoriasis” and combinations of these terms. The literature search mainly covered studies published between 2015 and 2026, while seminal studies establishing the fundamental biology of VISA/MAVS signaling were also included regardless of publication date. The final literature search was performed in March 2026.

Publications were selected based on their relevance to the regulation of VISA/MAVS signaling, with particular emphasis on studies providing mechanistic insights into signalosome activation, propagation, termination, degradation, and maintenance of the resting state. Original research articles were prioritized, whereas review articles were primarily consulted to identify key studies and to ensure comprehensive coverage of the field. The authors independently evaluated the relevance and scientific quality of the included literature to minimize selection bias.

## Figures and Tables

**Table 1:** PTMs promote the aggregation of VISA.

Regulator	Types of PTMs	Sites
TRIM31 [35]	K63-linked ubiquitination	K10/311/461
Ube2N [36]	K63-linked ubiquitination	K7/297/500
SIRT3 [37]	Deacetylation	K7
PIAS3 [38]	SUMOylation	K461/500
p38 [39]	Phosphorylation	S186/220
OGT [40]	O-Glycosylation	S366
ZDHHC24 [41]	Palmitoylation	C46/79
ZDHHC4 [42]	Palmitoylation	C79
ZDHHC12 [43]	Palmitoylation	C79
ZDHHC7 [44]	Palmitoylation	C508

**Table 2:** Post-translational regulations inhibit the aggregation of VISA.

Regulator	Types of PTMs	Sites
USP10 [36]	Deubiquitination	K10/311/461
SENP1 [38,55]	DeSUMOylation	K461/500
APT2 [41]	Depalmitoylation	C46/79
SIRT5 [37]	Desuccinylation	K7
PRMT7 [56,57]	Monomethylation	R52
PRMT9 [58]	Methylation	R41/43
N/A [59]	Lactylation	N/A
WDR77 [60]	N/A	N/A
SAMHD1 [61]	N/A	N/A
TAX1BP1 [62]	N/A	N/A
MAVI1 [63]	N/A	N/A
NLRX1 [64]	N/A	N/A

**Table 3:** Ubiquitination drives the degradation of VISA.

Regulator	Types of PTMs	Sites	Functions
ASB3 [65]	K48-linked ubiquitination	K297	Proteasomal degradation
TRIM28 [66]	K48-linked ubiquitination	K7/10/371/420/500	Proteasomal degradation
TRIM25 [67]	K48-linked ubiquitination	K7/10	Proteasomal degradation
AIP4 [68,69,70]	K48-linked ubiquitination	K371/420	Proteasomal degradation
MARCH5 [71]	K48-linked ubiquitination	K7/500	Proteasomal degradation
RNF115 [72]	K48-linked ubiquitination	K500	Proteasomal degradation
RNF90 [73]	K48-linked ubiquitination	N/A	Proteasomal degradation
RNF146 [74]	K48-linked ubiquitination	N/A	Proteasomal degradation
MALT1 [22]	K48-linked ubiquitination	N/A	Proteasomal degradation
Smurf1 [75,76,77]	K48-linked ubiquitination	N/A	Proteasomal degradation
Smurf2 [78]	K48-linked ubiquitination	N/A	Proteasomal degradation
RNF5 [79]	K48-linked ubiquitination	K362/461	Proteasomal degradation
RNF5 [80]	K27-linked ubiquitination	K362/461	Autophagy degradation
MARCH8 [81]	K27-linked ubiquitination	K7	Autophagy degradation
RNF34 [82]	K27-linked ubiquitination	K297/311/348/362	Autophagy degradation

**Table 4:** Upstream regulators license the selective degradation of VISA.

Regulator	Types of PTMs	Sites	Functions
PCBP1/2 [68,69]	N/A	N/A	AIP4-mediated proteasomal degradation
TAX1BP1 [70]	N/A	N/A	AIP4-mediated proteasomal degradation
MST4 [75]	N/A	N/A	Smurf1-mediated proteasomal degradation
Ndfip1 [76]	N/A	N/A	Smurf1-mediated proteasomal degradation
OTUD1 [77]	N/A	N/A	Smurf1-mediated proteasomal degradation
PB1 [80]	N/A	N/A	RNF5-mediated autophagy degradation
HFE [83]	N/A	N/A	p62-mediated autophagy degradation
TNKS1/TNKS2 [74]	PARylation	E137	RNF146-mediated proteasomal degradation
ZDHHC3 [84]	Palmitoylation	N/A	Proteasomal degradation
c-Abl [85]	Phosphorylation	Y9/11/30/71	Autophagy degradation
NLK [86]	Phosphorylation	S121/212/258/329	Degradation

## Data Availability

No new data were created or analyzed in this study. Data sharing is not applicable to this article.

## Abbreviations

The following abbreviations are used in this manuscript:

Abbreviation	Full Name
APT2	Acyl-protein thioesterase 2
ATP13A1	ATPase 13A1
CARD	Caspase recruitment domain
DHX	DExH-box helicase
FZR1	Fizzy and cell division cycle 20-related protein 1
GSDMB	Gasdermin B
IFN	Interferon
IKK	IκB kinase
IRFs	Interferon regulatory factors
ISGs	Interferon-stimulated genes
MAVS	Mitochondrial antiviral-signaling protein
Met	Mesenchymal–epithelial transition factor (MET receptor)
NF-κB	Nuclear factor kappa B
NLR	NOD-like receptor
NLRP6	NLR family pyrin domain containing 6
NOD2	Nucleotide-binding oligomerization domain-containing protein 2
O-GlcNAc	O-linked β-N-acetylglucosamine
OGT	O-GlcNAc transferase
PAMP	Pathogen-associated molecular pattern
PARylation	Poly-ADP-ribosylation
PCSK9	Proprotein convertase subtilisin/kexin type 9
PFKFB3	6-Phosphofructo-2-kinase/fructose-2,6-bisphosphatase 3
PLK1	Polo-like kinase 1
PRMT	Protein arginine methyltransferase
PRR	Proline-rich region
PRR	Pattern-recognition receptor
PTMs	Post-translational modifications
PWWP3A	PWWP domain-containing protein 3A
RIG-I	Retinoic acid-inducible gene I
RLRs	RIG-I-like receptors
SENP1	SUMO-specific protease 1
SIRT	Sirtuin
SLE	Systemic lupus erythematosus
SUMO	Small ubiquitin-like modifier
TBK1	TANK-binding kinase 1
TLR	Toll-like receptor
TRAFs	Tumor necrosis factor receptor-associated factors
uORFs	Upstream open reading frames
UBL7	Ubiquitin-like protein 7
VISA	Virus-induced signaling adaptor
ZDHHC	Zinc finger DHHC-type palmitoyltransferase
ZNFX1	Zinc finger NFX1-type containing 1
